# Mr. Cusack

**Published:** 1861-11

**Authors:** 


MR. CUSACK.—This eminent practitioner died on the night of the 26th of September, 1861, at his residence in Merrion-square, in the seventy-fourth year of his age. He was the head of the profession of surgeons in Ireland, and for many years occupied the most distinguished position connected with it in Dublin. His death causes many vacancies. He was a Fellow of the Royal College of Surgeons, of which he had been more than once president; he was consulting surgeon of Steevens's Hospital, and of Swift's Hospital, a member of the Board of Superintendence of Hospitals, and on the death of Sir Philip Crampton he was appointed Surgeon in Ordinary to the Queen. He also occupied the position of Regius Professor of Surgery in Trinity College. Until near the time of his death he continued to discharge his duties at the hospitals, and to see patients at his house. An attack of bronchitis baffled the skill of his medical attendants—the most eminent men in the city. He was regarded as one of the greatest ornaments of the medical profession in Ireland, and was held in the highest esteem by the public.—*Lancet*, Oct. 5, 1861.

